# TeloSearchLR: an algorithm to detect novel telomere repeat motifs using long sequencing reads

**DOI:** 10.1093/g3journal/jkaf062

**Published:** 2025-04-02

**Authors:** George Chung, Fabio Piano, Kristin C Gunsalus

**Affiliations:** Department of Biology, New York University, New York, NY 10003, USA; Center for Genomics and Systems Biology, New York University, New York, NY 10003, USA; Department of Biology, New York University, New York, NY 10003, USA; Center for Genomics and Systems Biology, New York University, New York, NY 10003, USA; Department of Biology, New York University, New York, NY 10003, USA; Center for Genomics and Systems Biology, New York University, New York, NY 10003, USA

**Keywords:** telomere, telomeric repeat motif (TRM), novel telomere detection, ALT, genome assembly, long-read sequencing

## Abstract

Telomeres are eukaryotic chromosome end structures that guard against sequence loss and aberrant chromosome fusions. Telomeric repeat motifs, the minimal repeating unit of a telomere, vary from species to species, with some evolutionary clades experiencing a rapid sequence divergence. To explore the full scope of this evolutionary divergence, many bioinformatic tools have been developed to infer novel telomeric repeat motifs using repetitive sequence search on short sequencing reads. However, novel telomeric motifs remain unidentified in up to half of the sequencing libraries assayed with these tools. A possible reason may be that short reads, derived from extensively sheared DNA, preserve little to no positional context of the repetitive sequences assayed. On the other hand, if a sequencing read is sufficiently long, telomeric sequences must appear at either end rather than in the middle. The TeloSearchLR algorithm relies on this to help identify novel telomeric repeat motifs on long reads, in many cases where short-read search tools have failed. In addition, we demonstrate that TeloSearchLR can reveal unusually long telomeric motifs not maintained by telomerase, and it can also be used to anchor terminal scaffolds in new genome assemblies.

## Teaser

Telomeres are DNA sequences and proteins that mark the ends of chromosomes in eukaryotes. Thanks to sequencing technologies available today, we can begin to appreciate the diversity of telomeric sequences and the many mechanisms that maintain them in different organisms. Some sequencing techniques necessarily break DNA into very small fragments, which make the determination of telomere sequence and length difficult. Other sequencing techniques do not, and combined with the use of TeloSearchLR described in this work, we can determine both the sequence and the length of telomeric DNA directly from sequence data.

This article has an associated podcast which can be accessed at https://players.brightcove.net/1611106596001/default_default/index.html?videoId=6373818433112.

## Introduction

Replicative DNA polymerases lack the ability to synthesize the extreme 5′ end of double-stranded linear DNA. Because of this, repeated cycles of DNA replication would lead to a progressive loss of chromosome ends, a phenomenon known as the End Replication Problem (Watson 1972; Olovnikov 1973). Several solutions to this problem exist in nature, including covalently closed hairpin ends found in certain bacteria and phages (Deneke *et al*. 2000; Kobryn and Chaconas 2002; Hertwig *et al*. 2003; Huang *et al*. 2004, 2012; Moriarty and Chaconas 2009; Ramírez-Bahena *et al*. 2014), and DNA synthesis primed by covalently linked terminal proteins found in several virus families (Salas 1991; Caldentey *et al*. 1992, 1993; Hoeben and Uil 2013). In eukaryotes, the most common solution is the regulated addition of tandemly repeated sequences to the 3′ end by telomerase (Greider and Blackburn 1989), circumventing the End Replication Problem by allowing more of the complementary 5′ end to be replicated.

Telomerase is a protein–RNA complex that synthesizes telomeric repeats using a short template on the telomerase RNA (Blackburn 1992). Even though the telomerase RNAs across the tree of life are not particularly well conserved in size or in primary sequence, the template region—and thus the telomeric repeat motif (TRM)—appears surprisingly constant (Zakian 1995; Podlevsky and Chen 2016): TTTAGGG in most flowering plants (Fajkus *et al*. 2019; Song *et al*. 2019; Peska and Garcia 2020) and TTAGGG in most metazoans (Traut *et al*. 2007; Logeswaran *et al*. 2021) except insects where the motif is mostly TTAGG (Okazaki *et al*. 1993; Sahara *et al*. 1999; Vítková *et al*. 2005). Nevertheless, certain clades appear to experience an accelerated divergence in their TRMs. Species in the genus *Allium* (onions, garlic, and relatives) have a CTCGGTTATGGG motif (Fajkus *et al*. 2016; Fajkus *et al*. 2019). Similarly, TRMs in coleopteran (beetles) (Okazaki *et al*. 1993; Sahara *et al*. 1999; Frydrychová and Marec 2002; Prušáková *et al*. 2021) and hymenopteran (sawflies, bees, wasps, and ants) (Zhou *et al*. 2022) species have repeatedly diverged from the ancestral insect motif.

Determining the full diversity of TRMs can allow us to find specific molecular drivers behind this diversity. As such, many different strategies have been deployed to detect telomeric repeats across species—mostly based on hybridization assays or bioinformatic analyses. Hybridization-based assays use labeled DNA probes to anneal to telomeric sequences: being the gold standard for verifying known telomeric motifs, especially in combination with the Bal-31 exonuclease (De Lange and Borst 1982), these assays are not suitable for identifying new and divergent TRMs (Prušáková *et al*. 2021), because generating probes for every possible sequence permutation that can appear at telomeres is impractical. Thus, bioinformatic strategies have been more successful at identifying novel TRMs.

Because telomeres maintained by telomerase are tandem repeat clusters of short (5–30 bp) motifs, most bioinformatic strategies rely on tandem repeat detection, using Tandem Repeats Finder (Benson 1999), mreps (Kolpakov *et al*. 2003), the Telomeric Repeats Identification Pipeline (Zhou *et al*. 2022), or a similar algorithm to find repeat clusters on short sequencing reads generated on the Illumina sequencing platform. These strategies rank the tandem repeats by their occurrence in the sequencing library. In species with relatively long telomeres, the telomeric motif is often one of the most frequent tandem repeats. Indeed, this strategy has successfully identified novel TRMs in many species of fungi (Červenák *et al*. 2021; Peska *et al*. 2021), plants (Peška *et al*. 2017), insects (Prušáková *et al*. 2021; Zhou *et al*. 2022), and nematodes (Lim *et al*. 2023).

These short-read search strategies are not without limitations. When TRMs are long—such as the one from *Kluyveromyces lactis* (25-bp long)—short read searches may fail because too few repeat copies are present on short sequencing reads (Peska *et al*. 2021). Furthermore, because these search strategies look for repeat motifs with the most frequent occurrences on sequencing reads, extremely short telomeres with very low repeat copy numbers may also be missed. Perhaps due to these and other limitations, a search strategy using Tandem Repeats Finder could not detect the TRM in 23 out of 96 Coleopteran species surveyed (Prušáková *et al*. 2021), and a similar search strategy using the Telomeric Repeats Identification Pipeline failed to detect the TRM in 66 out of 129 insect species surveyed (Zhou *et al*. 2022).

To fill in the gaps in the survey of telomeric motif diversity, we developed TeloSearchLR (Telomere Search on Long Reads), a new TRM search strategy that circumvents the limitations of the older search strategies by taking advantage of a growing number of publicly available long-read genomic sequencing libraries. We successfully used the algorithm to identify telomere repeat motifs where they were previously unknown. We also found that TeloSearchLR could reveal telomeric motifs even with a limited number of sequencing reads at as low as 5× genome coverage. Where the TeloSearchLR strategy failed to reveal telomeric motifs, the most likely cause was the telomeres being longer than the sequencing reads. Finally, our results indicate that TeloSearchLR can also be used to identify subtelomeric repeat sequences present on long sequencing reads.

## Methods and materials

### Algorithm design and key parameters

TeloSearchLR is a Python script that surveys long genomic sequencing reads for telomeric repeats. Long sequencing reads generated by either the Pacific Biosciences platform (PacBio) or the Oxford Nanopore Technologies (ONT) platform capture longer repeating motifs than short sequencing reads. Moreover, the lack of extensive DNA fragmentation in long-read sequencing preserves natural DNA ends—such as replication intermediates, DNA repair intermediates, or telomeres. Intact telomeres always appear in their canonical orientation (usually the “G-rich strand”) at the 3′ ends of long sequencing reads, and as their reverse complement (usually the “C-rich strand”) at the 5′ ends—a phenomenon we call “terminal stranded occupancy”. Using this logic, the TeloSearchLR algorithm helps us search for bona fide telomere repeat motifs (TRMs) by plotting the motif occupancy patterns at the 5′ and the 3′ ends of sequencing reads.

**Fig. 1.**
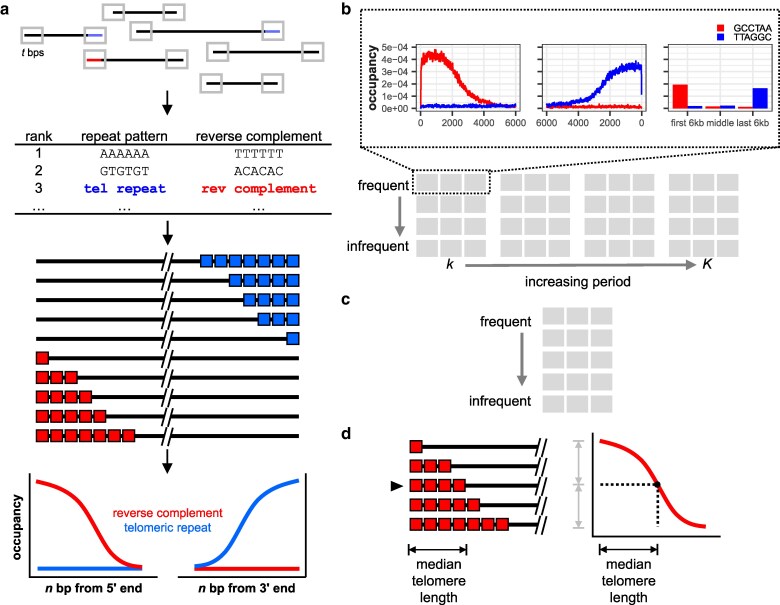
Algorithm design and the expected output. a) The TeloSearchLR algorithm examines the terminal *t* bp of long-read sequences for tandem repeats and ranks the repeat motif classes by how frequently they appear in a sequencing library. As natural dsDNA ends, TRMs (TRMs) are expected to be among the most abundant repeat classes and should show a strand bias: the conventional motif orientation (pointing toward the chromosome end) should be enriched at the 3′ ends of sequencing reads, while the reverse complement should be enriched at the 5′ ends. Visual inspection of the terminal stranded occupancy pattern should distinguish TRMs from other repeat motifs. b) Algorithm output in “exhaustive” mode. Occupancy patterns are graphed by distance from the 5′ and 3′ ends of sequencing reads and by terminal vs middle regions. Patterns are arranged by repeat motif period in columns, and by total occupancy in rows. c) Algorithm output in “occupancy” mode, which ranks repeat motifs by frequency irrespective of their period. d) The occupancy pattern naturally reflects the distribution of telomere lengths, such that the *x* value (bp) at half the maximum *y* value (occupancy) should reflect the median telomere length. Thus, telomere lengths may be estimated from the algorithm output.

TeloSearchLR involves two major steps: ranking and plotting. In the ranking step, TeloSearchLR uses TideHunter (v1.5.5) (Gao *et al*. 2019) to identify all tandem repeat motifs appearing within the first and the last *t* bp on all reads 2*t* bps or longer (default *t* = 1000, specified by the -t flag, Fig. 1a). The user specifies the range of repeat periods (in bps) for the algorithm to consider, using the -k (shortest period) and the -K (longest period) flags. TideHunter then produces a tabular output detailing the detected repeat motifs and their locations on the sequencing reads. Using this tabular output, TeloSearchLR generates a list of motifs ranked from the most frequent to the least frequent (Fig. 1a).

In the plotting step, TeloSearchLR iterates through the list of ranked motifs and determines the positions where each motif and its reverse complement are occupying on the sequencing reads, this time using the tabular output of a TideHunter search across entire reads (rather than just the terminal *t* bps). TeloSearchLR will plot the occupancy patterns for the most frequent motif (numerical ranking specified by the -m flag) to the least (-M flag followed by a number). The user also specifies a plotting window using the -n flag so that TeloSearchLR records the positions occupied by repeat motifs in the first and last *n* nucleotides as well as the middle of all sequencing reads 2*n* bp or longer. At the end of the plotting step, the putative TRMs can then be identified visually by their terminal stranded occupancy—where the reverse complement of the TRM is almost exclusively found at the first *n* bp of the reads, and the TRM is almost exclusively found at the last *n* bp of the reads (Fig. 1a). As read lengths are variable, the region excluding the first and last *n* bp is also variable. Thus, the occupancy fraction of a repeat motif in the middle of the reads is represented in a separate bar graph, and a true TRM should be underrepresented in the middle of sequencing reads (Fig. 1b).

TeloSearchLR can output the plots by the repeat motif period *and* by the motif occupancy (the “exhaustive mode”, enabled by the -e flag, Fig. 1b) or by occupancy only (the “occupancy mode”, by default, Fig. 1c). Sorting the plots by repeat period is useful when searching for repeat motifs of a known length, while sorting by occupancy is useful when the telomeric repeat period is unknown. Because the TRM occupancy plot reflects the distribution of telomere lengths, we can estimate the median telomere length by determining the *x* value at which the corresponding occupancy value (*y*) is roughly one-half of the maximum repeat occupancy on the same plot (Fig. 1d). The run commands for every library examined in this report are provided in Table 1, and the TRMs found by our algorithm, as well as the estimated median telomere lengths for various species, are reported in Table 2.

**Table 1.** Commands for TeloSearchLR analysis.

Organism	Run commands	Notes
*Anthonomus grandis*	python3 TeloSearchLR.py -f SRR16132310.fasta -k 4 -K 50 -m 1 -M 40 -t 1000 -n 4000 -e	
*Caenorhabditis elegans*	python3 TeloSearchLR.py -f SRR7594465.fasta -k 4 -K 20 -m 1 -M 100 -t 1000 -n 6000	
*Candida albicans*	python3 TeloSearchLR.py -f SRR7874309.fasta -k 4 -K 30 -m 1 -M 100 -t 1000 -n 3000	
*Capsicum chinense*	python3 TeloSearchLR.py -f SRR23734611.fasta -k 4 -K 20 -m 1 -M 100 -t 1000 -n 4000	
*Diabrotica virgifera*	python3 TeloSearchLR.py -f ERR6907800.fasta -k 4 -K 20 -m 1 -M 100 -t 1000 -n 800	
*Diadromus collaris*	python3 TeloSearchLR.py -f SRR6513331.fasta -k 4 -K 50 -m 1 -M 40 -t 1000 -n 4000 -e	
*Geotrupes spiniger*	python3 TeloSearchLR.py -f ERR11673226.fasta -k 4 -K 50 -m 1 -M 40 -t 1000 -n 4000 -e	
*Hyposoter dolosus*	python3 TeloSearchLR.py -f ERR11843429.fasta -k 4 -K 20 -m 1 -M 100 -t 1000 -n 5000	
*Kluyveromyces lactis*	python3 TeloSearchLR.py -f ERR10466726.fasta -k 4 -K 30 -m 1 -M 100 -t 1000 -n 1500	
*Magnusiomyces capitatus*	python3 TeloSearchLR.py -f SRR23362189.fasta -k 4 -K 20 -m 1 -M 100 -t 1000 -n 1500	
*Poecilus cupreus*	python3 TeloSearchLR.py -f ERR10812864.fasta -k 4 -K 20 -m 1 -M 100 -t 1000 -n 4000	
*Saccharomyces cerevisiae*	python3 TeloSearchLR.py -f SRR12427674.fasta -k 4 -K 20 -m 1 -M 100 -t 1000 -n 800	
*Sitophilus oryzae*	python3 TeloSearchLR.py -f Sitophilus_oryzae_ont.fasta -k 4 -K 20 -m 1 -M 100 -t 1000 -n 1000	Sitophilus_oryzae_ont.fasta is the concatenation of SRR9852240 to SRR9852244 libraries
*Sorghum bicolor*	python3 TeloSearchLR.py -f ERR4312692.fasta -k 4 -K 20 -m 1 -M 100 -t 1000 -n 4000 python3 TeloSearchLR.py -f SRR26636576.fasta -k 4 -K 20 -m 1 -M 100 -t 1000 -n 40000	
*Strongyloides stercoralis*	python3 TeloSearchLR.py -f SRR25177361_and_SRR25177362.fasta -k 4 -K 20 -m 1 -M 100 -t 1000 -n 8000 python3 TeloSearchLR.py -f SRR25177361_and_SRR25177362.fasta -k 21 -K 1000 -m 1 -M 100 -t 2000 -n 8000	SRR25177361_and_SRR25177362.fasta is the concatenation of SRR25177361 and SRR25177361 libraries.
*Vespa velutina*	python3 TeloSearchLR.py -f ERR3313355.fasta -k 4 -K 20 -m 1 -M 100 -t 1000 -n 10000	

**Table 2.** TRMs identified visually from terminal stranded occupancy compared to telomeric repeats identified by molecular and older bioinformatic methods.

			Published evidence							Long-read analysis (this work)			
	Species or genera	Published motif*^a^*	molecular cloning	terminal fragment Digest + Southern	dot blot	fluorescent in-situ hybridization	short-read library repeat analysis	inference from telomerase RNA	highly contiguous Genome assembly	TeloSearchLR-identified motif	median telomere length estimate (bp)	library run accession	sequencing technology
Well-characterized telomeric repeats	*Caenorhabditis elegans*	TTAGGC	*^b^*							TTAGGC	2500	SRR7594465*^c^*	PacBio
	*Candida albicans*	ACGGATGTCTAACTTCTTGGTGT	*^d^*	*^d^*						ACGGATGTCTAACTTCTTGGTGT	1000	SRR7874309*^e^*	ONT
	*Kluyveromyces lactis*	ACGGATTTGATTAGGTATGTGGTGT	*^f^*	*^f^*						ACGGATTTGATTAGGTATGTGGTGT	500	ERR10466726	ONT
	*Vespa velutina*	TTGCGTCAGGG							*^g,h^*	TTGCGTCAGGG	7000	ERR3313355*^i^*	PacBio
Variable repeats	*Saccharomyces cerevisiae*	(TG)_1–4_G_2-3_	*^j,k^*	*^k^*				*^l,m^*		(TG)_1–4_G_2-3_	300	SRR12427674*^n^*	ONT
	*Magnusiomyces capitatus*	T_1–2_A_3–5_G_3–8_						*^o^*	*^o^*	T_1–2_A_3–6_G_4–6_	500	SRR23362189	PacBio
Telomere repeat patterns previously missed by other assays and algorithms	*Diabrotica virgifera*	–T̶T̶A̶G̶G̶, T̶C̶A̶G̶G̶					*^p^*			TTAGG	200	ERR6907800*^q^*	PacBio
	*Poecilus cupreus*	–T̶T̶A̶G̶G̶, T̶C̶A̶G̶G̶			*^p^*					TTAGG	2250	ERR10812864*^g^*	PacBio
	genus *Sitophilus^r^*	–T̶T̶A̶G̶G̶, T̶C̶A̶G̶G̶			*^p^*					TTTGG	250	SRR9852240 to SRR9852244*^s^*	ONT
	genus *Hyposoter^r^*	?					*^t^*			TTTGGGTTTG	3000	ERR11843429*^u^*	PacBio
	genus *Geotrupes^r^*	–T̶T̶A̶G̶G̶		*^v^*						?	?	ERR11673226*^g^*	PacBio
	*Anthonomus grandis*	–T̶T̶A̶G̶G̶, T̶C̶A̶G̶G̶					*^p^*			?	?	SRR16132310	PacBio
	*Diadromus collaris*	?					*^t^*			?	?	SRR6513331	PacBio
Long telo-meres	*Capsicum chinense*	TT[T/C]AGGG				*^w^*	*^w^*	*^w^*		TT[T/C]AGGG	?	SRR23734611*^x^*	PacBio
	*Sorghum bicolor*	TTTAGGG							*^y^*	TTTAGGG	15000	SRR26636576	ONT (ultralong)
ALT?	*Strongyloides sterocoralis*	?					*^z^*			105-bp, 106-bp, and 111-bp repeats	?	SRR25177361 and SRR25177362	ONT

Patterns detected in *D. virgifera*, *P. cupreus*, *S. stercoralis*, and in genera *Sitophilus* and *Hyposoter* are novel candidate motifs identified using TeloSearch-LR.

*^a^* Patterns with a strikethrough indicate repeat motifs that have been ruled out previously. *^b^* Wicky *et al*. (1996). *^c^* Yoshimura *et al*. (2019). *^d^* McEachern and Hicks (1993). *^e^* Panthee *et al*. (2018). *^f^* McEachern and Blackburn (1994). *^g^* Darwin Tree of Life Project Consortium (2022). *^h^* Lukhtanov and Pazhenkova (2023). *^i^* Shi *et al*. (2019). *^j^* Szostak and Blackburn (1982). *^k^* Shampay *et al*. (1984). *^l^* Singer and Gottschling (1994). *^m^* Förstemann and Lingner (2001). *^n^* Barbitoff *et al*. (2021). *^o^* Brejová *et al*. (2019). *^p^* Prušáková *et al*. (2021). *^q^* Pest Genomics Initiative—Diabrotica virgifera (2024). *^r^* Previously tested species were *Hyposoter didymator, Sitophilus granrius, and Geotrupes stercorarius.* Species tested for this work are *Hyposoter dolosus, Sitophilus oryzae, and Geotrupes spiniger*. *^s^* Parisot *et al*. (2021). *^t^* Zhou *et al*. (2022). *^u^* Broad *et al*. (2024). *^v^* Frydrychová and Marec (2002). *^w^* Závodník *et al*. (2023). *^x^* Liu *et al*. (2023). *^y^* Paterson *et al*.(2009). *^z^* Lim *et al*. (2023).

We provide one additional way of running TeloSearchLR we named “single-motif mode”, enabled by the -s flag followed by the repeat motif. In this mode, TeloSearchLR only graphs the occupancy of a single repeat motif supplied by the user, thus skipping the ranking step altogether. This is useful for quickly checking if a specific motif is telomeric. Required parameters are the fasta read file (-f), the sequence motif to be examined (-s), the graphing window (-n), and a TideHunter tabular output file (-T), which can be from an earlier run of TeloSearchLR, or from a TideHunter run independent from TeloSearchLR.

### Sequencing libraries

Sequencing libraries were downloaded from the NCBI Sequence Read Archive (https://www.ncbi.nlm.nih.gov/sra) using the fasterq-dump command from SRAtoolkit (v3.0.5, https://github.com/ncbi/sra-tools/). Since sequencing quality information was not needed for TeloSearchLR, the libraries were downloaded in the FASTA format using the –fasta option. Table 2 lists the accession numbers and the relevant references for these libraries.

Where applicable, the coverage of sequencing libraries was calculated by mapping the reads to the published genomes of *Caenorhabditis elegans* (BioProject PRJNA13758, version WS295, available via WormBase) (Harris *et al*. 2020), *Hyposoter dolosus* (accession GCA_963921915.1) (Broad *et al*. 2024), and *Magnusiomyces capitatus* (accession GCA_030571335.1) (Opulente *et al*. 2023) using minimap2 v2.17 (Li 2018) and counting the mapped positions using samtools v1.14 (Danecek *et al*. 2021). The libraries were subsampled using seqtk v1.4 (Li 2024) to the desired coverage values.

## Results

### Detection of well-characterized TRMs

**Fig. 2.**
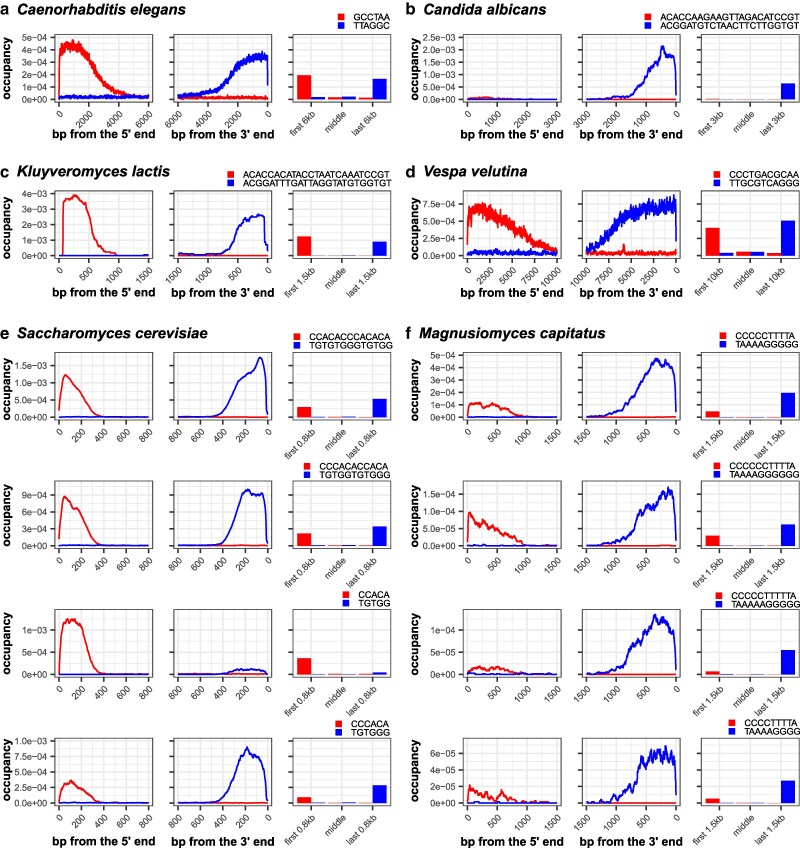
Detection of well-characterized telomere repeat motifs and variable telomeric repeat motifs. Occupancy patterns of TRMs (blue) and their reverse complements (red) for different species. a) TRM for the nematode *Caenorhabditis elegans*, TTAGGC. b) TRM for the yeast *Candida albicans*, ACGGATGTCTAACTTCTTGGTGT. c) TRM for the yeast *Kluyveromyces lactis*, ACGGATTTGATTAGGTATGTGGTGT. d) TRM for the wasp *Vespa velutina*, TTGCGTCAGGG. e) Top four most frequently observed TRMs for the brewing/baking yeast *Saccharomyces cerevisiae*. f) Top four TRMs for the yeast *Magnusiomyces capitatus*.

We benchmarked our algorithm on long genomic sequencing reads from organisms whose TRMs were previously identified by different methods. We aimed to demonstrate that our search strategy could detect a range of TRMs from four organisms—from 6-mer motifs (the nematode *C. elegans*) (Wicky *et al*. 1996) to 25-mer motifs (the yeast *Kluyveromyces lactis*) (McEachern and Blackburn 1994) (Table 2). The motifs of three species (*C. elegans*, *K. lactis*, and *Candida albicans*) were initially discovered through the molecular cloning of chromosome ends and verified with terminal restriction fragment digests (McEachern and Hicks 1993; McEachern and Blackburn 1994; Wicky *et al*. 1996). The motif for the wasp *Vespa velutina* was discovered through an examination (Lukhtanov and Pazhenkova 2023) of a highly contiguous genome assembly generated by the Darwin Tree of Life project (Darwin Tree of Life Project Consortium 2022). In all occupancy mode runs on *C. elegans*, *C. albicans, K. lactis,* and *V. velutina* long sequencing reads, the most frequent repeat motif with a clearly terminal stranded occupancy pattern is the previously identified TRM, confirming that TeloSearchLR can help us identify the TRM (Table 2, Fig. 2a-d, Supplementary Fig. 1-4). Several other repeat motifs also show a terminal and stranded occupancy pattern (Supplementary Fig. 1-4, pink boxes). These repeat motifs have a Hamming distance of 1 or 2 to the known TRM and appear at a much lower frequency: thus, these motifs likely represent sequencing errors or mutations to the telomeric sequences.

### Detection of telomeric motifs with variable repeat units

We next turned our attention to telomeric repeats motifs that are known to be variable. Several yeast species, including the widely studied *Saccharomyces cerevisiae* and the opportunistic pathogen *Magnusiomyces capitatus*, have telomeric repeat units that can vary in sequence and period due to the different possible ways to prime the extension of telomeric DNA using a telomerase RNA template (Förstemann and Lingner 2001) or the stalling/stuttering of the telomerase enzyme during telomere lengthening (Cohn and Blackburn 1995). TRMs in *S. cerevisiae* and *M. capitatus* were previously reported to be (TG)_1–4_G_2–3_ (Shampay *et al*. 1984) and T_1–2_A_3–5_G_3–8_ (Červenák *et al*. 2021) respectively. To detect telomeric repeats from these two species, we ran TeloSearchLR in the occupancy mode to plot the occupancy of the 100 most frequently occurring 4-mer to 20-mer repeats in the terminal regions of sequencing reads (-k 4 -K 20 -m 1 -M 100). In both species, several top ranked terminal stranded occupancy patterns broadly matched the TRMs previously reported—(TG)_1–4_G_2–3_ for *S. cerevisiae* and T_1–2_A_3–6_G_4–6_ for *M. capitatus* (Supplementary Fig. 5-6, red boxes). The occupancy plots of the top 4 terminally stranded motifs are shown in Fig. 2e-f.

In *S. cerevisiae,* we further detected a stranded occupancy pattern for the sequence AGGGCTATTT and its reverse complement AAATAGCCCT, located consistently ∼400 bps from the ends of sequencing reads (Supplementary Fig. 5, green box). Closer inspection revealed that these signals came from tandem repeats adjacent to the Y′ subtelomeric element in *S. cerevisiae* (Louis and Haber 1992). This demonstrated the potential for this algorithm to be used to detect sub-TRMs as well as TRMs.

### Detection of coleopteran and hymenopteran TRMs missed by dot blot, southern blot and short-read analysis

**Fig. 3.**
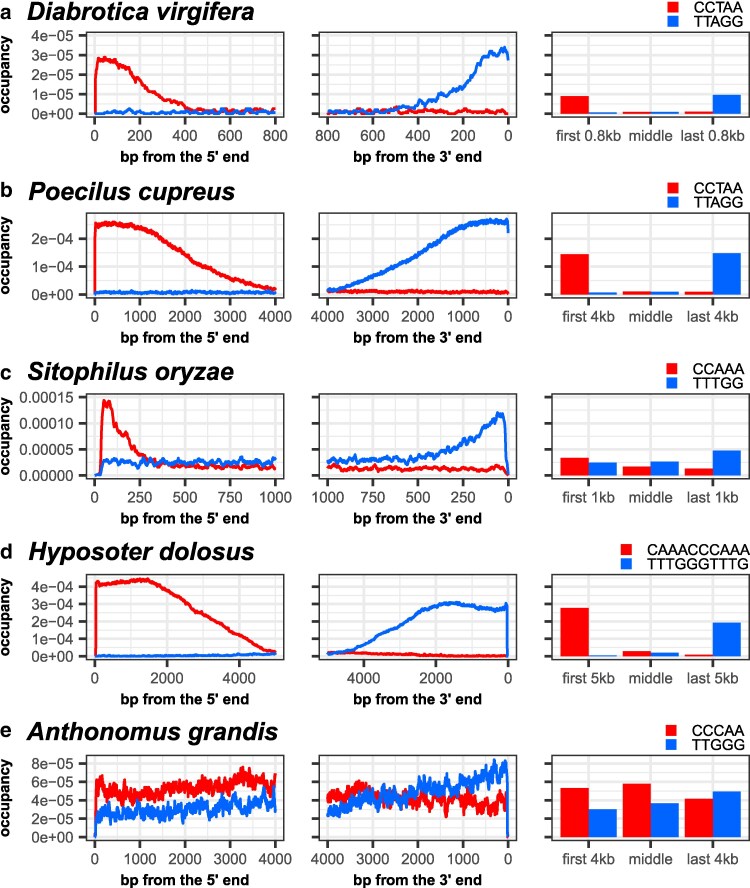
Detection of previously unidentified TRM. Occupancy patterns of TRM candidates (blue) and their reverse complements (red) for different species. a) Proposed TRM for the beetle *Diabrotica virgifera*, TTAGG. b) Proposed TRM for the ground beetle *Poecilus cupreus*, TTAGG. c) Proposed TRM for the rice weevil *Sitophilus oryzae*. d) Proposed TRM for the parasitoid wasp *Hyposoter dolosus*, TTTGGGTTTG. e) Candidate TRM for the boll weevil *Anthonomus grandis*, TTGGG. The occupancy patterns for this species do not show a strong strand bias.

Confident that our algorithm could help us detect novel TRMs, we tested it on long sequencing reads from the insect orders Coleoptera (beetles) and Hymenoptera (bees, wasps, and ants), where many species have divergent TRMs. In Table 2 and Fig. 3, we highlight 7 Coleopteran and Hymenopteran telomeric motifs missed by Southern blots, dot blots or short-read analysis (Frydrychová and Marec 2002; Prušáková *et al*. 2021; Zhou *et al*. 2022). Using occupancy plots generated by our algorithm, we detected stranded occupancy of the ancestral insect TTAGG telomeric motif in two species, *Diabrotica virgifera* and *Poecilus cupreus* (Fig. 3a-b, Supplementary Fig. 7-8), but a previous report ruled out this ancestral motif (Prušáková *et al*. 2021). We also successfully identified divergent TRMs in two genera (*Sitophilus oryzae—*TTTGG and *Hyposoter dolosus—*TTTGGGTTTG) where hybridization-based assays and short-read analyses failed to detect TRMs in other members of the same genera (Fig. 3c-d, Supplementary Fig. 9-10).

For *Anthonomus grandis*, *Geotrupes spiniger*, and *Diadromus collaris,* we could not detect any short (4–50 bp) motifs with clearly stranded occupancy patterns (Supplementary Fig. 11-13). For *G. spiniger*, even though the motif TTGGGG had a stranded occupancy pattern similar to bona fide telomeric motifs above (Supplementary Fig. 12, grey box), we ruled it out as the TRM for two reasons. First, this motif and its reverse complement were found only in the terminal ∼50 bp of reads—an extraordinarily short length for telomeres. Second, *both* CCCCAA and TTGGGG repeats were frequently found at opposite ends on the *same* reads, upon inspection. This was unexpected: unless these ∼10kb reads represented the entirety of extraordinarily small chromosomes, reads should only have *either* terminal TTGGGG repeats on the 3′ end *or* terminal CCCCAA repeats on the 5′ end, but not both (Fig. 1a). Thus, the telomeric motif of *G. spiniger* remains unidentified.

### Very long telomeres obscure the stranded occupancy of TRMs

**Fig. 4.**
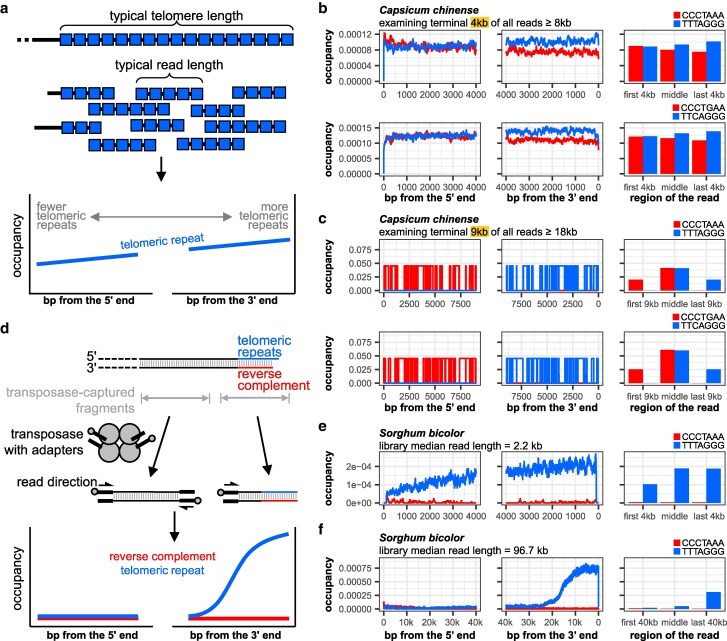
Known TRMs evade detection when telomeres are long. a) When the telomere is much longer than a typical sequencing read in a library, the TRM is no longer restricted to only one end of the read. Thus, the occupancy curve of the telomeric repeat does not show a stark strand bias. The reverse complement is omitted for clarity. b) Occupancy plots of the *Capsicum chinense* telomeric repeat, TT[T/C]AGGG, with a variant third nucleotide. c*) chinense* telomeres have been shown to be maintained by telomerase and two paralogous *TERC* copies differing at the T/C variant position, and both TTTAGGG and TTCAGGG probes hybridize to the chromosome ends (Závodník *et al*. 2023). Thus, we looked for the occupancy patterns of TTTAGGG and TTCAGGG repeats, considering all reads 8 kb or longer. c) For the same sequencing library, when only considering all reads 18 kb or longer, a stranded occupancy pattern emerges for TT[T/C]AGGG. d) One method of preparing “ultra-long” Nanopore sequencing library involves using a specialized transposase optimized for the attachment of sequencing adapters 50–100 kb apart. It is not possible to attach adapters on both ends of the most telomeric fragment, leading to these reads favoring the orientation with the telomeric repeats at the 3′ end. e) Occupancy plots of the *Sorghum bicolor* telomeric repeat, TTTAGGG, using a library with a median read length of 2.2 kb. f) Occupancy plots of the *S. bicolor* telomeric repeat, TTTAGGG, using an “ultra-long” sequencing library with a median read length of 96.7 kb.

Because our search strategy looks for motifs that are enriched at the 3′ or 5′ end of long sequencing reads, it would fail if the telomeres spanned longer than individual reads in the library. For *A. grandis*, we noticed that the occupancy patterns of TTGGG and its reverse complement CCCAA were slightly stranded (Fig. 3e, Supplementary Fig. 11 red box). Similarly in *G. spiniger*, the occupancy patterns for several motifs (TTGGG, TGACCA, TGACCT) were also slightly stranded (Supplementary Fig. 12 red boxes). These motifs are thus possible TRM candidates for these species, because when the sequencing reads are not long enough to capture entire telomeres, the counts of telomeric repeats could “spill over” from the last *n* nucleotides to the middle and the first *n* nucleotides of the reads (Fig. 4a), obscuring the clear strandedness seen in Fig. 1–3. Similarly, the counts of the reverse complement patterns would “spill over” to the last *n* nucleotides.

Supporting this idea, known TRMs sometimes do not have a clear terminal stranded occupancy pattern on long sequencing reads (Fig. 4a). For example, the TRM of the Habanero pepper *Capsicum chinense*, previously found to be TT[T/C]AGGG (Závodník *et al*. 2023), does not have an obviously stranded occupancy with the plotting window *n* = 4 kb, in a long-read library with a median read length of 13.5 kb (run accession SRR23734611) (Table 2, Fig. 4b and Supplementary Fig. 14). To see if the stranded occupancy pattern could be revealed by extending the plotting windows, we adjusted the *n* value from 4,000 to 11,000 (Supplementary Fig. 15a). However, this also had the effect of decreasing the number of reads being considered (Supplementary Fig. 15b), as fewer reads were 22,000 bps or longer (2 × 11,000 bps) in comparison with reads that were 8000 bps or longer (2 × 4000 bps) (Supplementary Fig. 15b). We could only detect a terminal stranded occupancy with *n* = 9000 or above (Fig. 4c, Supplementary Fig. 15c-j), but the median telomere length could not be estimated since too few telomeric reads were 18,000 bps or longer. This result indicates that *C. chinense* telomeres are often 9000 bp or longer. Our search strategy thus depends on genomic sequencing reads being much longer than typical telomeres in a biological sample.

### Detection of TRMs from long telomeres using ultra-long sequencing reads

Sequencing library preparation methods exist to generate “ultra-long” sequencing reads with lengths of up to 50–100 kb using specialized transposases to attach sequencing adapters and their preloaded sequencing helicases (Fig. 4d). For naturally long (>10 kb) telomeres, these ultra-long reads might reveal the terminal stranded occupancy patterns of the TRM. We found that the sorghum (*Sorghum bicolor*) motif, TTTAGGG (Paterson *et al*. 2009), did not have a typical terminal stranded occupancy from a library with a median read length of 2.2 kb (run accession ERR4312692) (Table 2, Fig. 4e, and Supplementary Fig. 16). However, in an ultra-long genomic library (run accession SRR26636576) with a median read length of 96.7 kb, we found the TTTAGGG TRM at the 3′ ends of the reads, but comparatively few CCCTAAA reverse complement repeats at the 5′ ends (Fig. 4f, Supplementary Fig. 17), with an estimated telomere median length of 15 kb.

The bias for telomeric repeats on the 3′ end may be due to the way these libraries were generated. Near the chromosome end, the transposase cannot attach an adapter at the extreme distal end due to the mechanism of transposition (Fig. 4d). Thus, these ultra-long telomeric reads would favor having telomeric repeats at the 3′ ends, rather than the reverse complement at the 5′ ends of the reads. The occupancy plots would not be mirrored, but instead show a more prominent occupancy curve for the 3′ motif than for its 5′ motif (Fig. 4, d and f), unlike most of the instances we have examined so far (Fig. 1–3).

### Detection of telomeric motifs with unusually long repeat periods maintained by telomerase-independent mechanisms

So far, we have focused on organisms that lengthen and maintain telomeres using telomerase—the predominant eukaryotic strategy to deal with the end replication problem; however, the telomerase gene has been lost in several eukaryotic lineages, with the most studied example being the Dipteran order (flies), where specialized transposons maintain telomere length (Mason *et al*. 2008). These telomerase-independent TRMs can be much longer than TRMs associated with the use of telomerase. Extending our algorithm to search for telomeres maintained by telomerase-independent mechanisms, we allowed the ranking step of TeloSearchLR to examine more than 1000 bp of the reads. This in turn allowed the algorithm to examine tandem repeats longer than 500 bp (as the longest *tandem* repeat motif to fit inside 1000 bp is 500 bp).

We chose to test our algorithm on the sequencing reads from the parasitic nematode *Strongyloides stercoralis*, for which a previous attempt to identify telomere repeat motifs using a short-read search strategy had been unsuccessful (Lim *et al*. 2023). Due to the simple karyotype (Kounosu *et al*. 2024) of 2*n* = 6, we reasoned *S. stercoralis* was the ideal organism to test, as there would only be 6 distinct chromosome ends to resolve in a homozygote. In addition, we were aided by a very contiguous reference genome assembly available for this species (GenBank accession GCA_029582065.1), with two autosomal scaffolds and several X chromosome scaffolds. It was unclear if the scaffold ends were fully resolved to reveal the true telomeres, so we aimed to identify all six telomeric ends in this species.

**Fig. 5.**
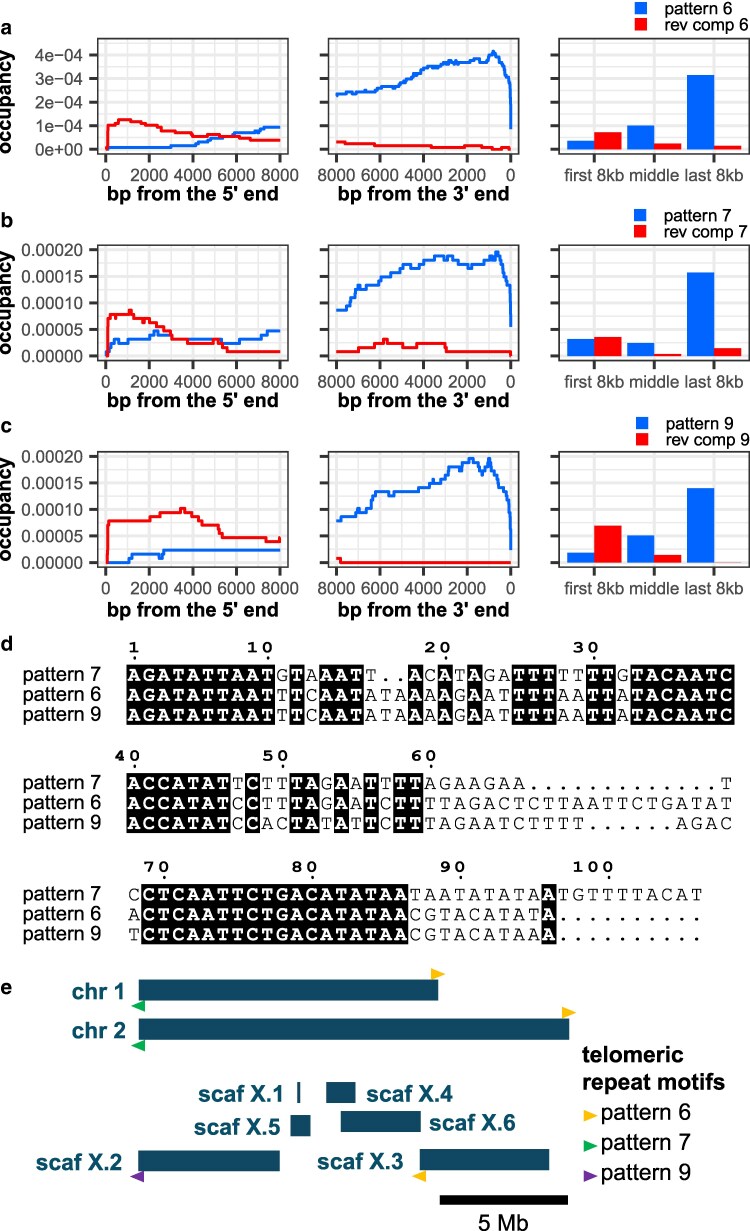
TRMs of *Strongyloides stercoralis.* Occupancy patterns of top three stranded *S. stercoralis* repeat motifs (blue) and their reverse complements (red). a) Repeat motif #6, a 111-mer. b) Repeat motif #7, a 106-mer. c) Repeat motif #9, a 105-mer. d) Multiple sequence alignment of motifs 6, 7, and 9. e) The locations of telomeres with motif 6 (yellow), 7 (green), and 9 (purple) in the genome assembly GCA_029582065.1. Arrowheads above the chromosome or scaffold schematic indicate the telomeric motifs in their conventional orientation (pointing toward the end of the chromosome), while those below indicate motifs represented by their reverse complement in the assembly. Scaffolds X.2 and X.3 are likely terminal scaffolds of the X chromosome.

Our search strategy did not turn up any short repeat motifs (4–20 bps) with a terminal stranded occupancy (-k 4 -K 20 -t 1000, Supplementary Fig. 18), so we examined tandem repeat motifs up to 1000 bps long, ranked by occupancies in the terminal 2000 bps on reads longer than 4000 bps (-k 21 -K 1000 -t 2000). Out of the top 100 repeat motifs (-m 1 -M 100), three repeat motifs and their reverse complements had terminal stranded occupancy, albeit with a noticeable “spillover effect” indicating that these telomeres are long. These three motifs are repeat pattern #6 (a 111-mer), #7 (a 106-mer), and #9 (a 105-mer) (Fig. 5a-c, Supplementary Fig. 19). The three motifs share significant sequence similarity and likely descended from a common sequence (Fig. 5d), and the presence of such long motifs at the telomere appears consistent with a type of alternative lengthening of telomeres (ALT) employed by nematodes and elsewhere (Seo *et al*. 2015; Kim *et al*. 2016, 2021; Lee *et al*. 2022; Stevens *et al*. 2022). We used the TideHunter output to extract reads with patterns #6, #7, and #9 and found that they mapped to the assembled scaffold ends (Fig. 5e, Supplementary Fig. 20, and see Supplementary Notes). Thus, we propose that these scaffold ends are the true telomeres of *S. stercoralis*, and we propose that the two X chromosome telomeres are located on X chromosome scaffolds X2 and X3.

### Identification of TRMs with low-coverage sequencing libraries

**Fig. 6.**
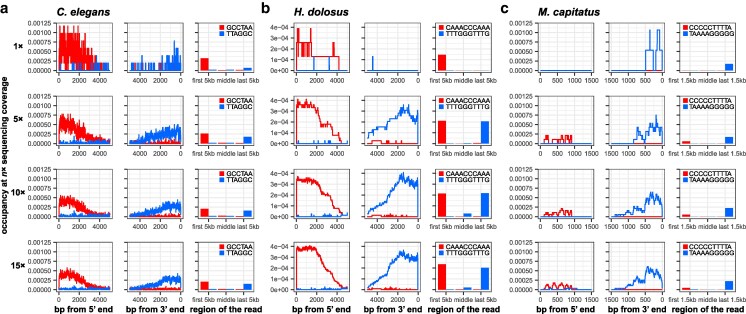
The effect of sequencing coverage on the stranded occupancy of telomeric repeats. a) The occupancy patterns of the *C. elegans* TRM TTAGGC with the reads subsampled to 1×, 5×, 10×, and 15×. b) The occupancy patterns of the *H. dolosus* TRM TTTGGGTTTG with the reads subsampled to 1×, 5×, 10×, and 15×. c) The occupancy patterns of the *M. capitatus* TRM TAAAAGGGGG with the reads subsampled to 1×, 5×, 10×, and 15×. In all cases, the stranded occupancy can be clearly observed starting from 5×.

Having limited amounts of material for a sequencing project is a common problem. To understand how low sequencing coverage could affect the search for a telomeric motif, we subsampled *C. elegans* (Yoshimura *et al*. 2019)*, M. capitatus, H. dolosus* (Broad *et al*. 2024) libraries down from 180×, 87×, and 398× genome coverage respectively to 1×, 5×, 10× or 15× genome coverage. We reliably detected telomeric motifs with 5× coverage and above (Fig. 6, Supplementary Fig. 21-23). With this coverage threshold lower than that required for assembling genomes (Sims *et al*. 2014), we believe TeloSearchLR can be readily added to existing genome assembly protocol workflows.

### Validation of telomere length estimates

We compared several of our telomere length estimates with published estimates to see if ours were in line with estimates based on Southern blots and various short-read repeat analyses (Table 3). This comparison is not perfect because the biological isolates used for long-read sequencing libraries are not identical to those used in separate studies. Nevertheless, we found that except for *C. elegans*, our telomere length estimates were broadly in line with previous estimates.

**Table 3.** Comparison of telomere length estimates.

	Previous estimates			Our estimates	
Species	Length (bp)	Strain assayed	Technique	Median length (bp)	Strain assayed
*Caenorhabditis elegans*	4,000 to 9,000*^a^*	N2	Southern	2500	VC2010/N2*^b^*
	2,000 to 9,000*^c^*	N2	Southern		
	4,120 to 83,700*^d^*	non-laboratory strains	TelSeq alg.		
*Candida albicans*	∼800*^e^*	WO-1	Southern	1000	TIMM1768*^f^*
	∼2,000*^g^*	BWP17	Southern		
*Kluyveromyces lactis*	250 to 500*^h,i^*	ATCC 32143	Southern	500	NCYC 416*^j0^*
*Saccharomyces cerevisiae*	250 to 400*^k^*	A364a, 2262	Southern	300	74-D694*^l^*
	200 to 300*^m^*	domesticated + wild strains	Y^ea^ISTY alg.		

*^a^* Wicky *et al*. (1996). *^b^* Raices *et al*. (2005). *^c^* Cook *et al*. (2016). *^d^* Yoshimura *et al*. (2019). *^e^* McEachern and Hicks (1993). *^f^* Singh *et al*. (2002). *^g^* Panthee *et al*. (2018). *^h^* McEachern and Blackburn (1994). *^i^* McEachern and Blackburn (1995). *^j^* SRA accession ERR10466726. *^k^* Walmsley and Petes (1985). *^l^* D’Angiolo *et al*. (2023). *^m^* Barbitoff *et al*. (2021).

## Discussion

We developed a telomere motif search strategy guided by TeloSearchLR, an intuitive algorithm whose graphical output allows the visual identification of TRMs at the ends of long sequencing reads (Table 2). We demonstrated that our search strategy was compatible with sequencing reads from both Pacific Biosciences (PacBio) or ONT platforms (Table 2), and that our strategy complemented existing telomere motif searches by successfully identifying novel TRMs that were missed (Table 2). Testing on a variety of different sequencing libraries, we found that our strategy succeeded when sequencing reads were much longer than typical telomeres in a biological sample. Moreover, we demonstrated that the median telomere length could be inferred directly from the occupancy plots generated by our algorithm.

### The use of repeat motif position allows the detection of TRMs from naturally short telomeres

Short-read TRM search strategies focus on the frequency of repeat motifs. In addition to frequency, TeloSearchLR also considers the position of the repeat motif occurrences, which is possible because long sequencing reads preserve natural DNA ends. Thus, an advantage to our approach over short-read search strategies is our ability to identify TRMs from naturally short telomeres, such as those from *D. virgifera* (median ≈ 200 bp). We suspect that the *D. virgifera* TRM eluded detection by short-read searches (Prušáková *et al*. 2021) because the TTAGG motif and its reverse complement were not heavily enriched in the sequencing library. In our analysis, TTAGG and its reverse complement represent only the 54th most frequent motif in the terminal 1000 bp of the *D. virgifera* sequencing reads (Supplementary Fig. 7), much lower ranked than TRMs in other species. Nevertheless, using TeloSearchLR we were able to conclude that TTAGG was the *D. virgifera* TRM by its terminal stranded occupancy on the sequencing reads. Thus, the consideration of repeat motif position on sequencing reads by TeloSearchLR provides a worthy addition to existing TRM search methods.

### TRMs from very long telomeres can be detected on even longer sequencing reads

When telomeres are of comparable or greater length than available sequencing reads, telomeric repeats are not enriched at opposite ends of the sequencing reads (Fig. 4a). We demonstrated that this limitation to our search strategy could be overcome partly by either filtering for very long reads (Fig. 4c) or by using sequencing libraries specifically designed to yield very long and intact DNA (Fig. 4f). In our analysis of the *S. bicolor* telomeres, a library with a median read length of 95 kb revealed the telomeres with a median length of 15 kb. Ultra-long reads also benefit genome assembly efforts as well, allowing the full resolution of long repetitive regions. Thus, we recommend the use of the longest possible sequencing read lengths for any novel sequencing project and for the identification of telomeres.

### TeloSearchLR can be used to anchor terminal scaffolds in new genome assemblies

Our strategy for identifying TRMs also complements new genome assembly efforts by anchoring the terminal scaffolds or contigs to their respective chromosome ends. The analysis of *Strongyloides stercoralis* sequencing reads revealed specific 105-bp, 106-bp, and 111-bp tandem repeats at the ends of sequencing reads: these helped anchored scaffolds X.2 and X.3 to the X chromosome ends (GenBank accession GCA_029582065.1). Thus, TeloSearchLR can be used as a quality checking step in the assembly of new genomes: when chromosome ends are fully resolved, they should include telomeres revealed by TeloSearchLR.

### Telomere lengths estimated from repeat occupancy plots may be shorter than other estimates

We expect our method to produce slightly shorter telomere length estimates compared to Southern blots. This is because our telomeric repeat occupancy tallies use “noisy” reads that are not completely error-free. While the TideHunter tandem repeat detection algorithm tolerates some sequencing errors (Gao *et al*. 2019), manual inspection of the TideHunter output revealed that telomeric repeat units with too many mismatches (likely from sequencing errors) were not counted (Supplementary Fig. 24a-b). This undercounting would lead to a shorter telomere length estimate (Supplementary Fig. 24c).

DNA library preparation methods also contribute to the underestimation of telomere length. Our source data come from typical genomic sequencing libraries that generally use end-blunting enzymes (and an A-tailing enzyme) to facilitate the attachment of double-stranded sequencing adapters (Supplementary Fig. 25a). These procedures are expected to eliminate 3′ single-stranded overhangs at telomeres. As a result, our algorithm only surveys the double-stranded portion of the telomere, whereas Southern blots can additionally reveal the single-stranded portion. Conceivably, the 3′ telomeric overhangs could be preserved with specialized library construction methods without the use of blunting exonucleases. The resulting reads would allow TeloSearchLR to reveal the full extent of the telomeric repeats, capturing both the double- and the single-stranded parts of the telomere, and allowing a comparison between this and the double-stranded telomere.

### A biased orientation places telomeric repeats at the 3′ end of sequencing reads

We initially expected the occupancy pattern of the TRM to mirror that of its reverse complement on the opposite ends of sequencing reads in all of the libraries we analyzed (Fig. 1a). However, motif occupancy patterns in some libraries are not mirrored, and most of these show a stronger preference for TRMs at the 3′ end of reads. We infer that technical differences in library preparation protocols, combined with specific characteristics of chromosome ends in some species, can give rise to biased attachment of sequencing adaptors to a specific end of a DNA sequence. For example, for the *C. albicans* (Fig. 2b) and *M. capitatus* (Fig. 2d) libraries, we hypothesize that non-B-DNA telomeric structures such as T-loops (de Lange 2004) and guanine quadruplexes (Schaffitzel *et al*. 2001; Biffi *et al*. 2013) are more resistant to DNA blunting and adapter ligation than standard B-form double-stranded DNA (Supplementary Fig. 25b-c). In the case of *S. bicolor* “ultra-long” reads (Fig. 4d), we attribute the asymmetry in telomeric motif enrichment to the use of a transposase during library preparation; we also found a similar asymmetry with ultra-long reads derived from humans (run accession DRR614096), cotton (run accession SRR26867867), and rice (run accession SRR27126942) (Supplementary Fig. 26a-c). All of these examples would lead to the preferential attachment of sequencing adapters at the nontelomeric end of terminal DNA fragments, leading to a lack of reads with the telomeric reverse complement at their 5′ ends.

### Extension of the algorithm to detect repetitive subtelomeres

Even though our method was specifically designed to detect TRMs, our analysis of the *S. cerevisiae* reads revealed a tandem repeat adjacent to the Y′ subtelomeric element. Similarly, we discovered several stranded occupancy patterns that were not terminal in *G. spiniger* but which could represent subtelomeric repetitive sequences (Supplementary Fig. 12, green boxes). This means that subtelomeric sequences, if they contain tandem repeats, can also be identified by our method.

In conclusion, we demonstrated that a novel telomere search strategy using TeloSearchLR could successfully identify novel telomeric repeats while providing an estimate of telomere length. When used in parallel while assembling a genome, TeloSearchLR provides a means to anchor telomeric genome scaffolds and to check the assembly ends for completeness. Moreover, our algorithm can be extended to identify subtelomeric repetitive elements. TeloSearchLR thus provides a valuable addition to existing tools for identifying telomere-associated repeat motifs.

## Supplementary Material

jkaf062_Supplementary_Data

## Data Availability

The TeloSearchLR.py source code is available via the TeloSearchLR repository on GitHub (https://github.com/gchchung/TeloSearchLR). Supplemental figures and material available on GSA FigShare: https://doi.org/10.25387/g3.28580036.
