# Supplementary material for: TeloSearchLR: an algorithm to detect novel telomere repeat motifs using long sequencing reads
Source: G3 (Bethesda). 2025 Apr 2;15(6):jkaf062. doi: 10.1093/g3journal/jkaf062 (PMC12134996; doi:10.1093/g3journal/jkaf062)
Supplement: jkaf062_Supplementary_Data [file jkaf062_supplementary_data.zip › Figure_S26 copy.pdf]

Figure S26

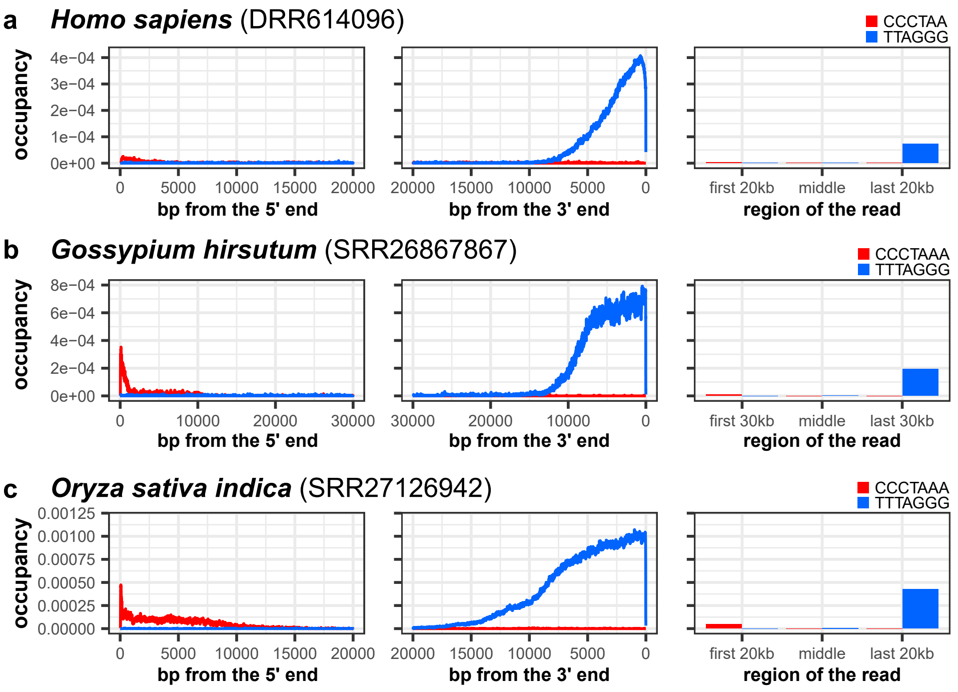

Figure S26: The biased read orientation places most telomeric repeats on the 3' end of ultra-long telomeric reads

a, The terminal stranded occupancy pattern of the human TRM, TTAGGG, and its reverse complement. **b,** The terminal stranded occupancy pattern of cotton (*Gossypium hirsutum*), TTTAGGG, and its reverse complement. **c,** The terminal stranded occupancy pattern of rice (*Oryza sativa indica*), TTTAGGG, and its reverse complement.
