# Supplementary material for: TeloSearchLR: an algorithm to detect novel telomere repeat motifs using long sequencing reads
Source: G3 (Bethesda). 2025 Apr 2;15(6):jkaf062. doi: 10.1093/g3journal/jkaf062 (PMC12134996; doi:10.1093/g3journal/jkaf062)
Supplement: jkaf062_Supplementary_Data [file jkaf062_supplementary_data.zip › supplemental_material_v20250313_for_resubmission.docx]

**Supplemental Materials for**

**TeloSearchLR: an algorithm to detect novel telomere repeat motifs using long sequencing reads**

George Chung* (gc95@nyu.edu), Fabio Piano, Kristin C Gunsalus* (kcg1@nyu.edu)

Department of Biology, New York University

Center for Genomics and Systems Biology, New York University

*Corresponding authors

## **KEYWORDS**

telomere, telomeric repeat motif (TRM), novel telomere detection, ALT, genome assembly, long-read sequencing

# **SUPPLEMENTAL NOTES**

## **Mapping *Strongyloides stercoralis* telomeric reads to the assembly**

In the *Strongyloides stercoralis* sequencing libraries (SRA run accessions SRR25177361 and SRR25177362), the three putative telomeric motifs found by TeloSearchLR are present at five of the six assembled chromosome ends in *S. stercoralis* (GenBank accession: GCA_029582065.1). The three motifs are named pattern 6, pattern 7 and pattern 9. The only chromosome end without one of these patterns was the right end of chr 1. This meant that the right end of chr 1 could be capped by a different (and potentially non-repetitive) sequence, or that this end was assembled incompletely or incorrectly.

To test if the right end of chr 1 was assembled correctly, we isolated reads with pattern 6, 7, and 9 repeats and mapped them to the assembly using minimap2 (Li 2018). Because TeloSearchLR results provided compelling evidence these were telomeric reads, any of these mapping to or near the right end of chr 1 could help us identify the true chromosome end. Reads with pattern 7 repeats mapped to the left ends of chr 1 and chr 2 (**Fig. S20a**). Reads with pattern 9 repeats mapped to the left end of scaffold X.2 (**Fig. S20a**). Reads with pattern 6 repeats mapped to the left end of scaffold X.3, the right end of chromosome 2, and to an interstitial region on chr 1, where alignments ended abruptly at position 11,637,827 roughly 35 kb from the assembled right end (**Fig. S20a**, arrowhead). For sequencing reads that map to this last location, instead of having parts of the ~35 kb sequence found in the assembly, these reads had a ~1490 bp of subtelomeric sequence before terminating in tandem repeats of pattern 6 (**Fig. S20b**). Thus, the ~35-kb sequence (11,637,828 - 11,673,123) at the right end of chr 1 was misassembled, and in its place should be the missing 1490-bp subtelomere and tandem repeats of pattern 6 as the telomere (**Fig. S20b**). We re-aligned the sequencing reads with pattern 6 repeats to this corrected configuration and found that they supported this chromosome end structure (**Fig. S20c**).

# **SUPPLEMENTAL FIGURES**

## **Figure S1: Occupancy plots of the 100 most frequently appearing 4- to 20-mer terminal repeat motifs in a *Caenorhabditis elegans* genomic sequencing library.**

The occupancy pattern for the *C. elegans* telomeric repeat motif, TTAGGC, is highlighted in red, while patterns for several telomere-repeat-like motifs are highlighted in pink.

## **Figure S2: Occupancy plots of the 100 most frequently appearing 4- to 30-mer terminal repeat motifs in a *Candida albicans* genomic sequencing library.**

The occupancy pattern for the *C. albicans* telomeric repeat motif, ACGGATGTCTAACTTCTTGGTGT, is highlighted in red, while patterns for several telomere-repeat-like motifs are highlighted in pink.

## **Figure S3: Occupancy plots of the 100 most frequently appearing 4- to 30-mer terminal repeat motifs in a *Kluyveromyces lactis* genomic sequencing library.**

The occupancy pattern for the *C. albicans* telomeric repeat motif, ACGGATTTGATTAGGTATGTGGTGT (as the cyclical equivalent ATTTGATTAGGTATGTGGTGTACGG), is highlighted in red.

## **Figure S4: Occupancy plots of the 100 most frequently appearing 4- to 20-mer terminal repeat motifs in a *Vespa velutina* genomic sequencing library.**

The occupancy pattern for the *V. velutina* telomeric repeat motif, TCAGGGTTGCG (as the cyclical equivalent GCGTCAGGGTT), is in red. The pattern for a telomere-like motif with 1 nucleotide difference is highlighted in pink.

## **Figure S5: Occupancy plots of the 100 most frequently appearing 4- to 20-mer terminal repeat motifs in a *Saccharomyces cerevisiae* genomic sequencing library.**

The occupancy patterns for the *S. cerevisiae* telomeric repeat motifs are highlighted in red. The stranded occupancy pattern of a tandem repeat (AGGGCTATTT) from the Y' subtelomeric element is highlighted in green.

## **Figure S6: Occupancy plots of the 100 most frequently appearing 4- to 20-mer terminal repeat motifs in a *Magnusiomyces capitatus* genomic sequencing library.**

Occupancy patterns for the *M. capitatus* telomeric repeat motifs are highlighted in red.

## **Figure S7: Occupancy plots of the 100 most frequently appearing 4- to 20-mer terminal repeat motifs in a *Diabrotica virgifera* genomic sequencing library.**

The occupancy pattern for the *D. virgifera* telomeric repeat motif candidate, TTAGG, is highlighted by the red box.

## **Figure S8: Occupancy plots of the 100 most frequently appearing 4- to 20-mer terminal repeat motifs in a *Poecilus cupreus* genomic sequencing library.**

The occupancy pattern for the *P. cupreus* telomeric repeat motif candidate, TTAGG, is highlighted by the red box. Patterns for possible subtelomeric repeat motifs are highlighted in green.

## **Figure S9: Occupancy plots of the 100 most frequently appearing 4- to 20-mer terminal repeat motifs in a *Sitophilus oryzae* genomic sequencing library.**

The occupancy pattern for the *S. oryzae* telomeric repeat motif candidate, TTTGG, is highlighted by the red box.

## **Figure S10: Occupancy plots of the 100 most frequently appearing 4- to 20-mer terminal repeat motifs in a *Hyposoter dolosus* genomic sequencing library.**

The occupancy pattern for the *H. dolosus* telomeric repeat motif candidate, TTTGTTTGGG, is highlighted in red.

## **Figure S11: Occupancy plots of the 40 most frequently appearing 4- to 50-mer terminal repeat motifs in an *Anthonomus grandis* genomic sequencing library, grouped by repeat period.**

The occupancy pattern for an *A. grandis* telomeric repeat motif candidate, TTGGG, is highlighted in red.

## **Figure S12: Occupancy plots of the 40 most frequently appearing 4- to 50-mer terminal repeat motifs in a *Geotrupes spiniger* genomic sequencing library, grouped by repeat period.**

The occupancy patterns of several slightly stranded repeat motifs are highlighted in red. The occupancy pattern of a possible subtelomeric repeat motif, TACCCA, is highlighted in green. Another stranded pattern, with the motif TTGGGG, has been ruled out as the telomeric repeat and is highlighted in grey.

## **Figure S13: Occupancy plots of the 40 most frequently appearing 4- to 50-mer terminal repeat motifs in a *Diadromus collaris* genomic sequencing library, grouped by repeat period.**

No occupancy patterns show clear strandedness in this library.

## **Figure S14: Occupancy plots of the 100 most frequently appearing 4- to 20-mer terminal repeat motifs in a *Capsicum chinense* genomic sequencing library.**

The occupancy patterns for the two known *C. chinense* telomeric repeat motifs, TTCAGGG and TTTAGGG, are highlighted by grey boxes.

## **Figure S15: Effects of increasing the plotting window length *n* on occupancy plots of repeat motifs in a *Capsicum chinense* genomic sequencing library.**

**a**, Graphical representation of changing the graphing window value *n*. The sequencing reads must be longer than or equal to 2*n* nucleotides long for the repeat occupancies to be counted. **b**, Distribution of sequencing read lengths for the sequencing library SRR23734611, with the y-axis in log scale to reveal the few very long reads. As the value of *n* increases, fewer sequencing reads will be 2*n* nucleotides or longer. **c**, The occupancy patterns of the two *C. chinense* telomeric repeat motifs, TTCAGGG and TTTAGGG, with a plotting window of *n* = 4000 bps. **d-j**, The occupancy patterns with *n* = 5000, 6000, 7000… 10000, 11000 bps. A terminal stranded occupancy pattern is apparent starting from *n* = 9000 (**h**).

## **Figure S16: Occupancy plots of the 100 most frequently appearing 4- to 20-mer terminal repeat motifs in a *Sorghum bicolor* genomic sequencing library.**

The occupancy pattern for the *S. bicolor* telomeric repeat motif candidate, TTTAGGG, is highlighted in grey.

## **Figure S17: Occupancy plots of the 100 most frequently appearing 4- to 20-mer terminal repeat motifs in a *Sorghum bicolor* ultra-long-read genomic sequencing library.**

The occupancy pattern for the *S. bicolor* telomeric repeat motif, TTTAGGG, is highlighted in red.

## **Figure S18: Occupancy plots of the 100 most frequently appearing 4- to 20-mer terminal repeat motifs in a *Strongyloides stercoralis* genomic sequencing library.**

The occupancy pattern for the *S. bicolor* telomeric repeat motif, TTTAGGG, is highlighted in red.

## **Figure S19: Occupancy plots of the 100 most frequently appearing 21- to 1000-mer terminal repeat motifs in a *S. stercoralis* genomic sequencing library.**

The occupancy patterns for the *S. stercoralis* telomeric repeat motif candidates, repeat #6, 7 and 9, are highlighted in red.

## **Figure S20: *Strongyloides stercoralis* sequencing reads with pattern 6, 7 and 9 repeats map to assembly ends.**

**a,** *S. stercoralis* genomic sequencing reads (SRA run accessions: SRR25177361 and SRR25177362) with pattern 6, 7 and 9 tandem repeats map to five assembly ends (Genbank accession GCA_029582065.1): reads with pattern 6 repeats map to chr 2 right and scaffold X.3 left; reads with pattern 7 repeats map to chr 1 left and chr 2 left; and reads with pattern 9 repeats map to scaffold X.2 left. Pattern 6 reads also map to a region near the right end of chr 1, but the alignments abruptly end at position 11,637,827, which suggests a misassembly event. **b,** Sequencing reads with pattern 6 repeats that map to chr 1: 11,637,827 do not agree with the assembled sequence. Instead of having parts of the ~35 kb sequence from 11,637,828 bp to the end of the assembly, the reads have a ~1490-bp subtelomeric sequence followed by tandem repeats of pattern 6. **c,** Sequencing reads with pattern 6 repeats support the corrected chr 1 end with the ~1490-bp sequence and pattern 6 tandem repeats.

## **Figure S21: The effect of sequencing coverage on the stranded occupancy of telomeric repeats in a *C. elegans* library.**

The 100 most frequent tandem repeat occupancy patterns and their reverse complements in reads subsampled to 1×, 5×, 10× and 15×. The occupancy patterns for the *C. elegans* TRM, TTAGGC, are highlighted in red.

## **Figure S22: The effect of sequencing coverage on the stranded occupancy of telomeric repeats in a *H. dolosus ns* library.**

The 100 most frequent tandem repeat occupancy patterns and their reverse complements in reads subsampled to 1×, 5×, 10× and 15×. The occupancy patterns for the *H. dolosus* TRM, TTTGGGTTTG, are highlighted in red.

## **Figure S23: The effect of sequencing coverage on the stranded occupancy of telomeric repeats in an *M. capitatus* library.**

The 100 most frequent tandem repeat occupancy patterns and their reverse complements in reads subsampled to 1×, 5×, 10× and 15×. The occupancy patterns corresponding to the *M. capitatus* variant TRM, T_1-2_A_3-6_G_4-6_, are highlighted in red.

## **Figure S24: Sequencing errors in the telomeric motif can lead to under-counting of repeat numbers and a shorter telomere length estimate.**

**a,** An idealized depiction of telomeric reads with the telomeric reverse complement motifs at the 5' ends of the reads. Red boxes indicate the position and the span of a telomeric repeat unit. **b,** In reality, not all of the repeat units are counted due to sequencing errors, indicated by the green ‘x’. **c,** As a result, median estimates of the telomeric length may be shorter (green) than the true median (red) due to noisy reads and under-counting of telomeric repeat units.

## **Figure S25: Typical long-read sequencing library construction and telomeric reads with strong sequencing strand bias.**

**a,** Standard sequencing library construction typically involves enzymatic blunting and A-tailing to the DNA to be sequenced. In the blunting step, the usual 3' telomeric overhang is expected to be deleted by the blunting enzyme. Thus, the single-stranded telomere sequence is lost in the sequencing step, leading to a shorter telomere length estimate by our method. **b,** The G-rich telomeric strand (blue) is prone to form guanine quadruplex structures under certain conditions. This may render the telomere end resistant to end repair and sequencing adapter attachment, leading to a sequencing strand orientation bias. **c,** The 3' telomeric overhang can form T-loops. This may render the telomere end resistant to end repair and sequencing adapter attachment, leading to a sequencing strand orientation bias.

## **Figure S26: The biased read orientation places most telomeric repeats on the 3' end of ultra-long telomeric reads**

**a,** The terminal stranded occupancy pattern of the human TRM, TTAGGG, and its reverse complement. **b,** The terminal stranded occupancy pattern of cotton (*Gossypium hirsutum*), TTTAGGG, and its reverse complement. **c,** The terminal stranded occupancy pattern of rice (*Oryza sativa indica*), TTTAGGG, and its reverse complement.

# **SUPPLEMENTAL REFERENCES**

Li H. 2018. Minimap2: pairwise alignment for nucleotide sequences. Bioinformatics. 34(18):3094–3100. doi:10.1093/bioinformatics/bty191.
