# Supplementary figures and images for: TeloSearchLR: an algorithm to detect novel telomere repeat motifs using long sequencing reads
Source: G3 (Bethesda). 2025 Apr 2;15(6):jkaf062. doi: 10.1093/g3journal/jkaf062 (PMC12134996; doi:10.1093/g3journal/jkaf062)

Figure S15

a

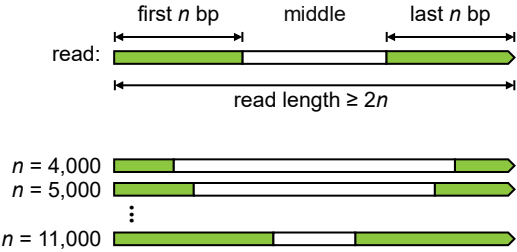

b

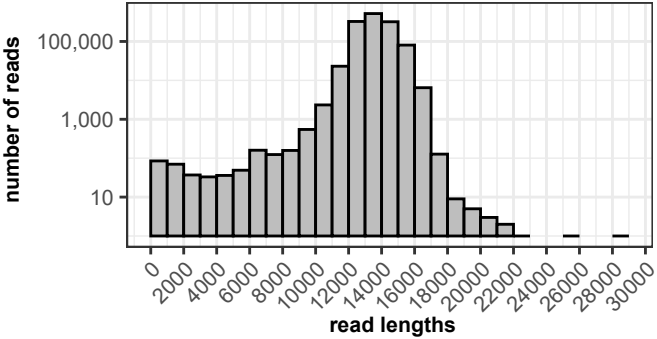

c

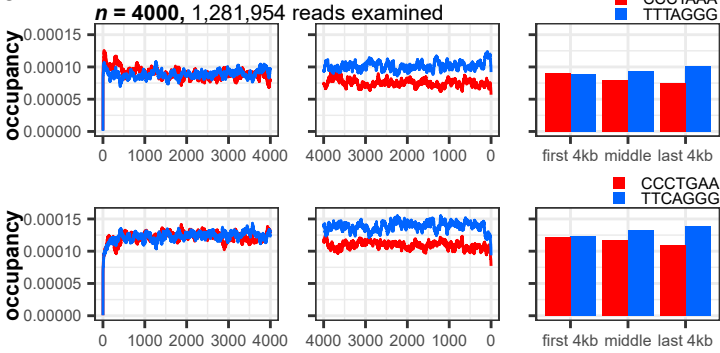

d

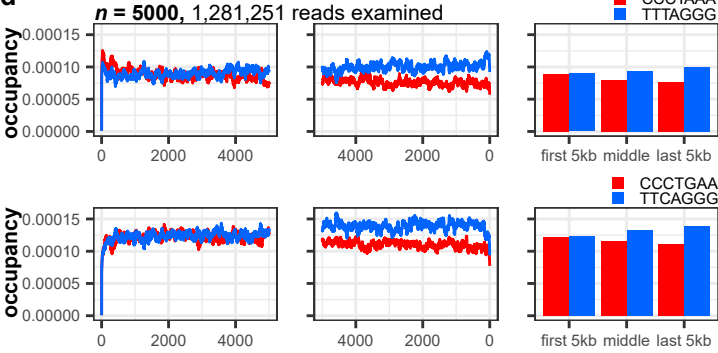

e

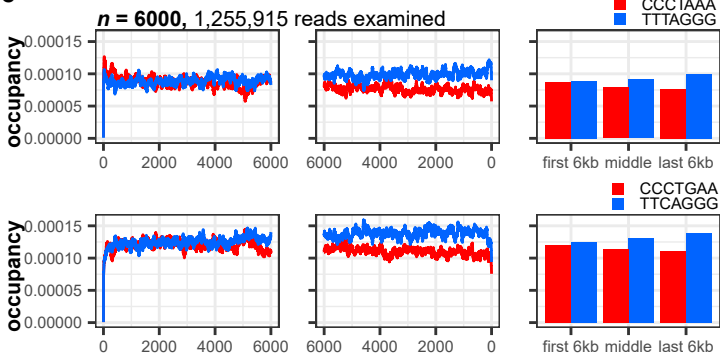

f

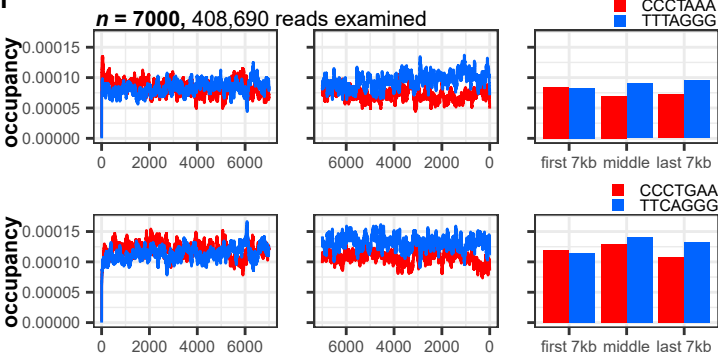

g

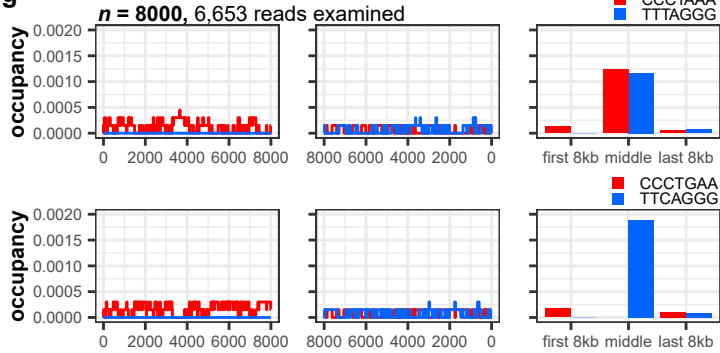

h

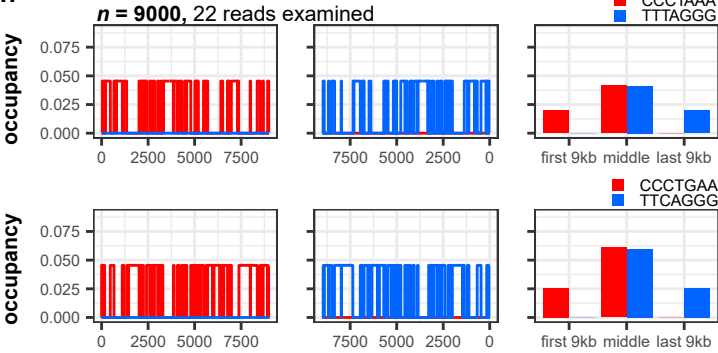

i

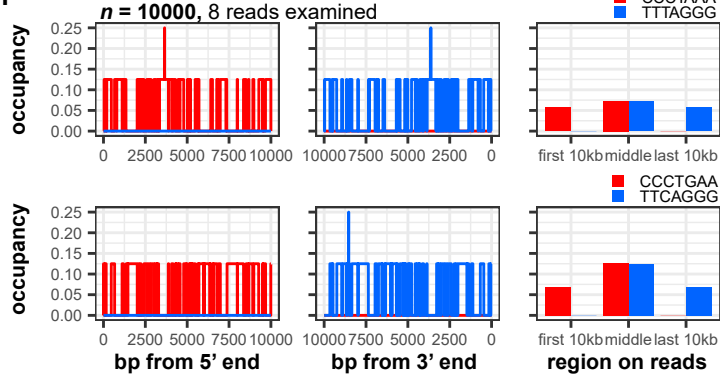

j

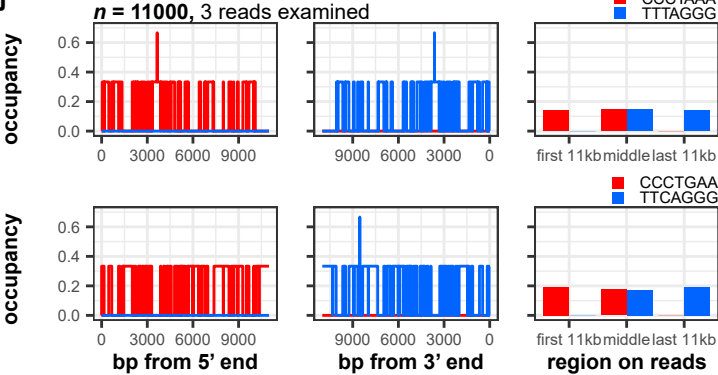

Supplement: jkaf062_Supplementary_Data [file jkaf062_supplementary_data.zip › Figure_S15 copy.pdf]

**Figure S20**

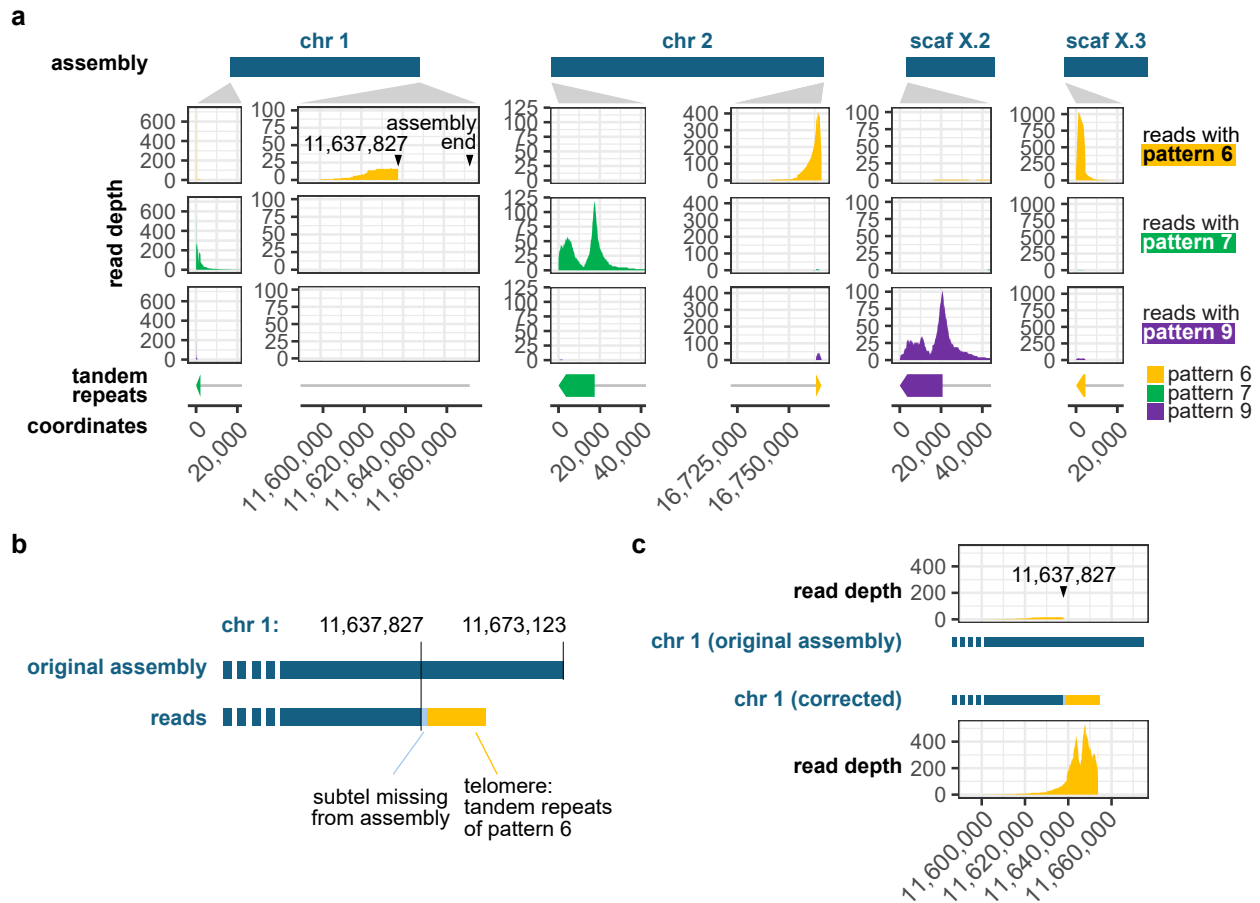

Supplement: jkaf062_Supplementary_Data [file jkaf062_supplementary_data.zip › Figure_S20_Strongyloides_stercoralis_misassembly_(label_adjust) copy.pdf]

Figure S24

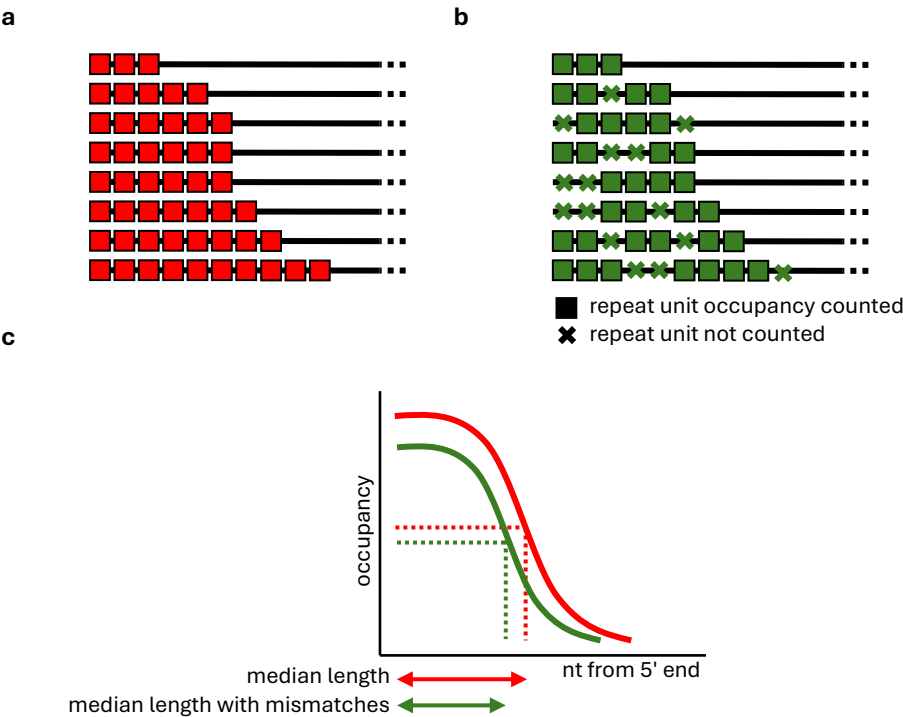

Supplement: jkaf062_Supplementary_Data [file jkaf062_supplementary_data.zip › Figure_S24 copy.pdf]

Figure S25

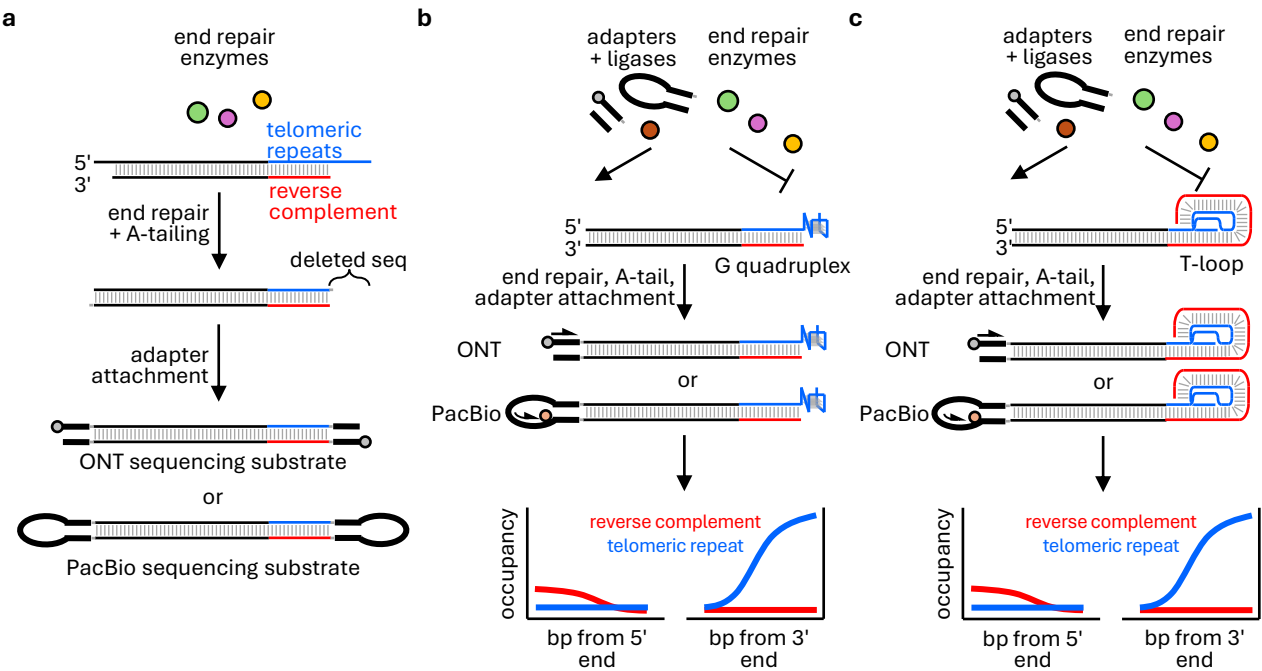

Supplement: jkaf062_Supplementary_Data [file jkaf062_supplementary_data.zip › Figure_S25 copy.pdf]
