# Supplementary material for: TeloSearchLR: an algorithm to detect novel telomere repeat motifs using long sequencing reads
Source: G3 (Bethesda). 2025 Apr 2;15(6):jkaf062. doi: 10.1093/g3journal/jkaf062 (PMC12134996; doi:10.1093/g3journal/jkaf062)

Figure S2

Candida albicans
SRR7874309

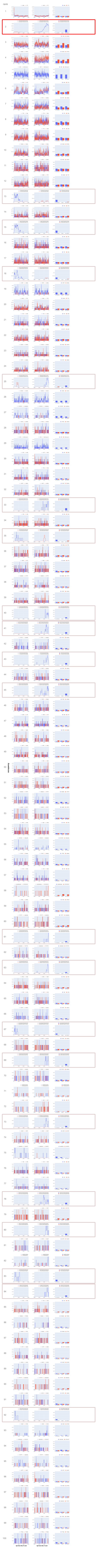

Supplement: jkaf062_Supplementary_Data [file Figure_S02_Candida_albicans copy.pdf › Figure_S02_Candida_albicans copy.pdf]
