# Supplementary material for: TeloSearchLR: an algorithm to detect novel telomere repeat motifs using long sequencing reads
Source: G3 (Bethesda). 2025 Apr 2;15(6):jkaf062. doi: 10.1093/g3journal/jkaf062 (PMC12134996; doi:10.1093/g3journal/jkaf062)
Supplement: jkaf062_Supplementary_Data [file Figure_S11_Anthonomus_grandis copy.pdf › Figure_S11_Anthonomus_grandis copy.pdf]

rank
1
2
3
4
5
6
7
8
9
10
11
12
13
14
15
16
17
18
19
